# Restrained Eating and Vegan, Vegetarian and Omnivore Dietary Intakes

**DOI:** 10.3390/nu12072133

**Published:** 2020-07-17

**Authors:** Anna Brytek-Matera

**Affiliations:** Institute of Psychology, University of Wroclaw, Dawida 1, 50-527 Wroclaw, Poland; anna.brytek-matera@uwr.edu.pl

**Keywords:** vegetarian diet, vegan diet, cognitive restraint, repetitive negative thinking, orthorexia nervosa

## Abstract

There are a significant number of studies on cognitive restraint among individuals with varying dietary patterns. Although most research has found that vegetarians report higher levels of cognitive restraint compared to non-vegetarians, many studies have contributed inconsistent results. The aim of the current study, therefore, was to assess any differences between groups with varying dietary patterns on cognitive restraint and other disordered eating pattern. The second objective was to examine determinants of cognitive restraint in individuals adhering to a vegan diet, a vegetarian diet and an omnivore diet. Two-hundred and fifty-four participants with varying dietary patterns completed the Three-Factor Eating Questionnaire, the Perseverative Thinking Questionnaire and the Eating Habits Questionnaire. Our results indicated that both vegetarian and vegan groups showed a significantly lower cognitive restraint, lower emotional eating and lower uncontrolled eating than those who followed an omnivorous diet. In addition, these both groups following a plant-based diet have shown more cognitions, behaviours and feelings related to an extreme focus on healthy eating (orthorexia nervosa) than group following an omnivorous diet. There were no significant differences between the groups in perseverative thinking. Core characteristics of repetitive negative thinking was a significant predictor of cognitive restraint in vegans. Feeling positively about healthy eating predicted cognitive restraint among vegetarians. Problems associated with healthy eating and feeling positively about healthy eating predicted cognitive restraint among individuals following an omnivorous diet. Knowledge of predictors of cognitive restraint may serve as a psychological intervention goal or psychoeducation goal among individuals with varying dietary patterns.

## 1. Introduction

A healthy vegetarian eating pattern provides recommendations to meet the dietary guidelines for those who follow a meat-free diet [1]. The healthy vegetarian eating pattern includes increased intake of legumes (beans and peas), soy products (particularly tofu and other processed soy products), nuts and seeds and whole grains. It contains no meats, poultry or seafood. In contrast to a vegetarian diet, a vegan diet excludes (a) animal products (eggs, dairy, beeswax and honey, leather products and goose-fat shoe polish); (b) garlic, onion, spring onion, scallions and leeks (c) products that may contain animal ingredients not included in their labels or which use animal products in their manufacturing, e.g., cheeses that use animal rennet (enzymes from animal stomach lining), gelatin (from animal skin, bones and connective tissue); (d) some sugars that are whitened with bone char (e.g., cane sugar but not beet sugar) [2].

The decision to adhere to a vegetarian diet is reported to be influenced by ethics reasons (e.g., moral considerations), health reasons (e.g., concern for potential disease, control of weight), concern about animal welfare (distaste for meat), preference for vegetarian food and/or religious and cultural beliefs [2,3,4].

Vegetarianism is restrictive by nature, regardless of any co-occurring eating pathology [5]. There are two distinct theories that explain dietary restraint [6]. The first, called the boundary model [7], explains how restrained eating can lead to unsuccessful dieting (dieting equals dietary restraint). Many diets fail due to the disinhibition of restraint effect (also known as the ‘what the hell’ effect). That is where a person is forced to violate her/his self-imposed restraint through eating forbidden foods or overconsuming calories (often in response to emotional distress) and continues to eat more than she/he usually would. In contrast, individuals who do not follow strict dietary rules tend to not have forbidden foods and when forced to overconsume on calories tend to compensate by eating less at the next meal. The second theory, a three-factor model of dieting behaviour [8] (frequency of dieting and overeating, current dieting and weight suppression) postulates that dietary restraint develops as the result of frequent dieting and overeating in the past rather than from current weight loss dieting or cognitive restraint. This model caused confusion in the restraint literature as to whether restraint is a measure of current restriction or a measure of dieting and overeating history [9]. Nowadays, it is generally accepted that dietary restraint is a construct that includes why a person is dieting (“restraint” is often explained as a long-term or habitual pattern of eating behaviour [9]), while dieting itself is the observed behaviour of restricting or intending to restrict, calories [6].

Cognitive restraint is defined as the mental effort applied to modify eating behaviours in order to maintain a restrictive dietary pattern [10]. In other words, it is the intention to control food intake in order to maintain or lose weight. Previous studies focusing on differences between vegetarians and omnivores have shown inconsistent results. Several studies have suggested higher cognitive restraint scores in vegetarians, others have found evidence for higher cognitive scores in non-vegetarians, and others have not found any differences between the two groups for this construct (Table 1).

Cognitive restraint is frequently used as a marker of pathological eating behaviours taking into consideration its consequences: dysregulation of internal perceptions of hunger and satiety, disinhibition which results in overeating, emotional dysregulation, low self-esteem and low body satisfaction [12].

Vegetarianism appears to be disproportionately associated with disordered eating [5]. However, the relationship is complex and the causality between cognitive restraint and vegetarianism is difficult to prove because: (a) current measures of cognitive restraint may inadequately measure restrictive behaviours in vegetarians and they could overestimate pathological eating behaviours in this group, (b) some vegetarians may score higher on measures of cognitive restraint because they restrict animal products on a daily basis, and not due to disordered eating behaviours [5].

One of the inappropriate eating behaviours among individuals with varying dietary patterns is orthorexia nervosa. This disordered eating pattern is characterized by a pathological fixation on consuming foods based on their quality (rather than their quantity) for achieving optimal health and/or avoiding illness [25,26]. Orthorexia nervosa is associated with avoidance of certain food groups (i.e., carbohydrates and fats), avoidance of foods with artificial substances and food prepared with the use of herbicides or pesticides [27], deleterious health behaviours (e.g., nutritional deficits, starvation) [28] and psychological consequences (e.g., social isolation, low quality of life, excessive fear and worries related to healthy dietary intake) [29,30]. Nowadays, there is a lack of consensus regarding the link between a vegetarian diet and orthorexia nervosa [31]. Nevertheless, it is worth pointing out that the latest literature review [31] have shown that vegetarian diet adherence was related to orthorexia nervosa (in 11 out of 14 studies published within the last five years). Furthermore, in seven studies (out of 14), vegetarians in the general population have been found to report more orthorexia nervosa or to be at risk of developing orthorexia nervosa than non-vegetarians.

Orthorexia nervosa shares several characteristics with vegetarianism, veganism and dieting behaviour (e.g., specific food selection-consuming healthy and organic food, focusing on quality of food intake, reduction of food intake according to specific nutrition rules, nutrition rules specifying which foods are “allowed” and which are “forbidden, rigid food rules and an inability to remain flexible in one’s eating habits) [19]. All of these dietary patterns include restrictions which resemble cognitive restraint. The continuous reduction of “allowed” foods results in a diet with only very few foods considered edible [16]. It is worth pointing out that recent study has shown that orthorexia nervosa can interfere with cognitive restraint [19] as well as that plant-based diet might be a contributing factor for the onset of orthorexia nervosa [16].

Extreme pursuit of healthy eating often lead to obsessive thoughts about food [32]. Repetitive negative thinking is a style of thinking about one’s problems (current, past or future) or negative experiences (past or anticipated) which are characterized by repetitiveness, intrusiveness and difficulty in disengaging from (the actual thinking process) [33]. In addition, repetitive negative thinking captures mental capacity and individuals perceive it as unproductive (individuals’ perceived dysfunctional effects of repetitive negative thinking). To the best of our knowledge, there is only one study examining the difference between vegetarians and non-vegetarians in the field [34]. Vegetarians showed more daily repetitive negative thinking than omnivores.

Although the perception of a relationship between vegetarianism and cognitive restraint remains widespread, there is presently no compelling empirical evidence to suggest that vegetarians are at greater risk of disordered eating behaviours compared to omnivores [5,35]. Therefore, the objectives of the present study were to: (1) investigate any differences between groups with varying dietary patterns on cognitive restraint and other disordered eating pattern; and (2) assess determinants of cognitive restraint among adults with varying dietary patterns (vegan, vegetarian and omnivore dietary styles).

On the basis of the literature, we put forward the following hypotheses:

**Hypothesis 1** **(H1).** *Individuals adhering to plant-based diet (vegan and vegetarian diet) have lower levels of cognitive restraint than those adhering to an omnivorous diet*.

**Hypothesis 2** **(H2).** *Individuals following a vegetarian and a vegan diet present a higher level of orthorexic behaviours compared to the individuals following a meat diet.*

**Hypothesis 3** **(H3).** *A disordered eating pattern (orthorexia nervosa) is a predictor of cognitive restraint among individuals following a vegetarian and vegan diet.*

**Hypothesis 4** **(H4).** *Repetitive negative thinking is a predictor of cognitive restraint among individuals following a vegetarian and vegan diet.*

Results of the present study could contribute to identifying potential factors for cognitive restraint and to gain a better insight into dietary patterns in general.

## 2. Materials and Methods

### 2.1. Participants and Study Design

Internet-based surveys are often used in psychology and health research (e.g., [36]). To collect the data, an online survey (SurveyMonkey^®^) was created and used. Participants were recruited through: (a) flyers distributed to local health food stores, vegan or vegetarian restaurants, (b) fitness centers, (c) universities and (d) through the internet (e.g., vegan or vegetarian social networking). Of the 308 individuals who began the questionnaires, finally 254 (82.46%) completed the survey and met the inclusion criteria (47 participants following a vegan diet, 100 participants following a vegetarian diet and 107 participants following an omnivorous diet). Eligibility criteria included both inclusion and exclusion criteria and comprised age (at least 18 years of age); consistency of self-defined types of diet (a “yes/no” item) and objective criteria for vegetarianism (exclusion of animal-derived foods such as meat, poultry and fish from the diet), veganism (exclusion of all animal products from the diet including dairy, cheese and eggs; a diet based solely on plant-based food) and an omnivorous diet (inclusion of animal and plant foods); and following a vegetarian or vegan diet for at least 12 months. All participants who met the inclusion criteria listed above were selected for the present study. Participants who answered in the affirmative were asked a number of other questions regarding the type of vegetarian diet, reasons for beginning and maintaining the diet and length of time the diet has been followed. Participants (5.84%, *N* = 18) were excluded from the study because of a discrepancy between self-identification and objective criteria (e.g., those who described themselves as vegetarians and declared sometimes eating meat or fish were eliminated). The procedure was presented in our previous publication [37]. The present study has been approved by a research ethics committee (No. WKEB45/03/2017). All procedures performed in our study were in accordance with the 1964 Helsinki declaration (adopted by the 18th World Medical Association General Assembly, Helsinki, Finland) and its later amendments or comparable ethical standards. The research project was funded by the National Science Centre (NCN), Poland (Grant no. 2017/01/X/HS6/00007). The current study was part of a large project focusing on the assessment of repetitive negative thinking and eating behaviours in daily life among individuals with varying dietary patterns.

### 2.2. Outcome Measures

Eating behaviours (The Three-Factor Eating Questionnaire [37]), repetitive negative thinking (the Perseverant Thinking Questionnaire [33]) as well as cognitions, behaviours and feelings related to orthorexia nervosa (the Eating Habits Questionnaire [38]) were measured in the present study (Table 2). All questionnaires used in the present study were formatted using a commercial online survey utility provided by Survey Monkey^®^.

In addition, the participants were asked questions regarding age, anthropometry (weight and height), health and lifestyle status (type of special diet, level of physical activity) and eating habits (number of meals consumed per day, daily water intake, dietary supplement consumption and type of supplement consumed, main reason why participants decided to follow a plant-based diet and main reason why participants are following a plant-based diet as well). All this information was obtained using a questionnaire specially designed for this study (the statistics presented in Table 3 are based on replies obtained in this questionnaire).

Intensity of physical activity (moderate- and vigorous-intensity physical activity) was based on the World Health Organization’s (WHO) recommendation on physical activity for adults aged 18–64 [41]: at least 150 min of moderate-intensity physical activity throughout the week and at least 75 min of vigorous-intensity physical activity throughout the week. In addition, the number of hours spent on different physical activity levels were obtained and converted into metabolic equivalents (METs). Average METs for moderate physical activity is equal to 4.0 and for vigorous physical activity is equal 8.0. Using these values, the scores expressed as MET-minutes/week were defined as: (a) moderate MET-minutes/week = 4.0 (intensity level) * moderate-intensity activity minutes (duration of activity) * moderate days (frequency of activity) and (b) vigorous MET-minutes/week = 8.0 * vigorous-intensity activity minutes * vigorous-intensity days [42]. Moderate-intensity physical activity was obtained by taking into account 5 or more days of any combination of walking, moderate-intensity or vigorous intensity activities achieving a minimum total physical activity of at least 600 MET-minutes/week. However, vigorous-intensity physical activity was computed by using 7 or more days of any combination of walking, moderate-intensity or vigorous-intensity activities achieving a minimum total physical activity of at least 3000 MET-minutes/week [42].

Due to the nature of the study (a web-based survey), types of physical measurements (height, weight and body mass index (BMI)) were self-reported (we were unable to invite participants to take direct measurements and use standard laboratory measurement, e.g., dual-energy X-ray absorptiometry, bioelectrical impedance analysis). BMI was calculated for self-reported values.

## 3. Results

### 3.1. Statistical Analysis

Statistical analyses were carried out using the Statistical Package for Social Sciences 25.0 (SPSS, Chicago, IL, USA). Associations with categorical variables (independent variable: dietary patterns; dependent variables: eating behaviours, repetitive negative thinking, orthorexia nervosa) were investigated with one-way analysis of variance (ANOVA) (comparison between participants following a vegan diet, vegetarian diet and omnivorous diet). Partial *η*2 was calculated as measure of effect size. Associations with continuous variables were examined with Pearson’s r correlation coefficients (relationship between cognitive restraint and orthorexia nervosa and repetitive negative thinking across the dietary patterns). To predict cognitive restraint a multiple linear regression analysis was used (predictors of cognitive restraint among individuals with varying dietary patterns).

### 3.2. Characteristics of the Study Population

Descriptive characteristics of the participants are presented in Table 3.

No significant between-group difference was observed in terms of age, *F* (2,251) = 0.85, *p* = 0.425, *η*^2^ = 0.007; as well as body mass index, *F* (2,251) = 2.66, *p* = 0.071, *η*^2^ = 0.021. However, statistical significance was found for a following plant-based diet, *t* (154) = 2.37, *p* = 0.019, Cohen’s *d* = 0.45.

### 3.3. Comparison between Participants Following a Vegan Diet, Vegetarian Diet and Omnivorous Diet: One-Way Analysis of Variance

The results of the one-way ANOVA with dietary patterns (vegan diet, vegetarian diet and omnivorous diet) as an independent variable and the Three-Factor Eating Questionnaire dimensions as the outcome indicates that there is a significant group difference in cognitive restraint, *F* (2,251 = 12.28, *p* < 0.001, *η*^2^ = 0.089; emotional eating, *F* (2,251) = 15.07, *p* < 0.001, *η*^2^ = 0.107 and uncontrolled eating, *F* (2,251) = 28.32, *p* < 0.001, *η*^2^ = 0.184. In addition, our finding shown that there is a significant group difference in the Eating Habits Questionnaire dimensions linked to problems related to healthy eating, *F* (2,251) = 23.67, *p* < 0.001, *η*^2^ = 0.59; knowledge of healthy eating, *F* (2,251) = 35.93, *p* < 0.001, *η*^2^ = 0.223 and feeling positively about healthy eating, *F* (2,251) = 8.20, *p* < 0.01, *η*^2^ = 0.61. Whereas, there are no significant differences between the groups in the dimensions of the Perseverative Thinking Questionnaire namely: core features of repetitive negative thinking, *F* (2,251) = 0.34, *p* = 0.709, *η*^2^ = 0.03; unproductive of repetitive negative thinking, *F* (2,251) = 1.02, *p* = 0.362, *η*^2^ = 0.008 and capturing mental capacity, *F* (2,251) = 0.37, *p* = 0.688, *η*^2^ = 0.003 (Table 4).

To sum up, individuals following a plant-based diet have a significantly lower levels of cognitive restraint, lower levels of emotional eating and lower levels of uncontrolled eating than individuals following an omnivorous diet. However, they are more likely to engage in cognitions, behaviours and feelings related to an extreme focus on healthy eating (orthorexia nervosa) compared to individuals following an omnivorous diet.

### 3.4. Relationship between Cognitive Restraint and Orthorexia Nervosa and Repetitive Negative Thinking Across the Dietary Patterns: The Pearson Correlation Coefficient

The structural relationships between cognitive restraint and orthorexia nervosa and repetitive negative thinking are presented in Table 5.

In vegans, cognitive restraint is related to core characteristics of repetitive negative thinking and cognitions related to orthorexia nervosa. In vegetarians, cognitive restraint is correlated to cognitions, behaviours and feelings related to orthorexia nervosa. While, in individuals adhering to an omnivorous diet cognitive restraint is associated with all dimension of repetitive negative thinking and all dimension of orthorexia nervosa.

### 3.5. Predictors of Cognitive Restraint among Individuals with Varying Dietary Patterns: α Multiple Linear Regression

In order to investigate determinants of cognitive restraint among adults with varying dietary patterns a multiple linear regression analysis was used (Table 6).

As shown in Table 6, higher levels of repetitiveness, intrusiveness and difficulties to disengage from repetitive negative thinking predict cognitive restraint in vegans. Positive feelings related to orthorexia nervosa predict cognitive restraint in vegetarians. However, problems associated with healthy eating and feeling positively about healthy eating predict cognitive restraint in individuals following an omnivorous diet.

## 4. Discussion

The current study was designed to determine whether differences in cognitive restraint and other disordered eating patterns exist between individuals adhering to plant-based diet (vegan and vegetarian diet) and those adhering to an omnivorous diet.

Our results demonstrated that both vegetarians and vegans endorse significantly lower cognitive restraint, lower emotional eating and lower uncontrolled eating in comparison to those who followed an omnivorous diet. These results confirm our hypothesis (H1). Our findings are similar to previous study founding that restricting food intake for purposes of weight control was significantly higher in individuals following an omnivorous diet compared with those following a vegetarian diet [12,15,18]. In non-vegetarians, cognitive restraint could counteract the effects of overconsuming (attempts to control intake may be activated by the desire to undertake weight-loss dieting and, therefore, higher restraint may be a marker for the adverse appetitive traits or overeating tendencies), whereas in vegetarians and vegans, the overeating tendency is nearly absent and cognitive restraint may results in eating less (because they are used to restricting the amount and quality of food consumed) [9,12]. Vegetarians and vegans appear to have the healthiest attitudes towards food. However, we still do not know whether or not a vegetarian diet could actually serve as a protective factor against developing disordered eating [35]. It may be assumed that higher restraint scores in non-vegetarians can be interpreted as indications of disordered eating or maladaptive attitudes towards food. 

Our results are consistent with a previous study [35] showing that vegans and vegetarians scored significantly lower than non-vegetarians on external eating. In our study, non-vegetarians showed higher emotional eating than vegetarians and vegans. This could potentially means that they learned to associate food and mood and then craved food when in a low-mood state or they could use food as a coping mechanism during low mood [6]. The second explanation could be that eating a favourite food had the propensity to release endogenous opioids and finally elevated their mood [6]. In our explanation the most dominant theory on emotional eating, the Macht five-way model [43], can be also useful. This model describes five way that explain how emotions affect eating: (1) emotions aroused by food stimuli affect food choice (emotions alter food choice through initiating cravings); (2) emotions high in arousal or intensity suppress eating (due to incompatible emotional responses); (3) emotions moderate in arousal or intensity affect eating depending on motivations to eat (in emotional eating, negative emotions elicit the tendency to be regulated by eating and, as a consequence, enhance intake of sweet and high-fat foods); (4) emotions consume cognitive resources, therefore individuals who use cognitive resources to govern what to eat will struggle to maintain those rules under emotional load; (5) emotions provide a feedback loop and interact with food to increase or decrease its pleasantness depending on the strength and orientation of the mood [43].

In addition, our findings show that both groups following a plant-based diet show more cognitions, behaviours and feelings related to an extreme focus on healthy eating defined as orthorexia nervosa (greater problems related to healthy eating, greater knowledge of healthy eating and greater positive feelings regarding healthy eating) than groups following an omnivorous diet. This suggests that individuals with orthorexia nervosa tendencies are more likely to be on a vegetarian and a vegan diet. Hypothesis 2 was confirmed. Previous studies have demonstrated similar results using the same questionnaire (the Three-Factor Eating Questionnaire) [12,37,42]. Other studies (using other methods assessing orthorexia nervosa) have also found that vegetarians and vegans were more likely than omnivores to display orthorexia nervosa [16,44,45]. Adhering to plant-based diets could prompt more focus on the quality of food (main core of orthorexia nervosa) and food consumption which may indicate that vegetarians and vegans are more likely to suffer from orthorexia nervosa or be at risk of developing orthorexia nervosa [15]. It is worth pointing out that previous studies found that vegetarians tend to demonstrate a higher level of nutrition knowledge than non-vegetarians [46] and showed a greater desire for more, improved information to apply to their eating habits [47]. However, as DeMay et al. [48] states, most data were based on vegetarian-related questions. Preliminary evidence suggests that vegetarians are generally more health-focused and vegans are more likely to be health-conscious than omnivores [49]. Many vegetarians follow healthy lifestyle patterns in greater frequency than non-vegetarians [50], however in some individuals, interest in healthy attitudes and behaviors towards food may show obsessive signs [19] and leads to orthorexia nervosa.

Our findings showed that vegetarians, vegans and non-vegetarians do not differ in perseverant thinking. Thus, there is no difference between these groups in core characteristics of repetitive negative thinking, unproductiveness of repetitive negative thinking and the degree to which repetitive negative thinking captures mental capacity. This would suggest that these three groups presented a similar level of repetitive negative thinking.

Our findings show that in vegans, cognitive restraint was associated with core characteristics of repetitive negative thinking and cognitions related to orthorexia nervosa. Cognitive restraint prevents food intake that is related to negative emotional content [51] and can lead to increased negative thoughts. Because both orthorexia nervosa and cognitive restraint shares a key component -self-imposed restriction of allowed food [12], it is not surprising that evidence has been found for a link between cognitive restraint and orthorexia nervosa in individuals following a plant-based diet (in vegetarians, cognitive restraint is correlated to cognitions, behaviours and feelings related to orthorexia nervosa). In individuals adhering to an omnivorous diet cognitive restraint was associated with all dimension of repetitive negative thinking and all dimension of orthorexia nervosa. This indicates that increased mental effort applied to modify eating behaviours in order to maintain a restrictive dietary pattern is related to higher levels of repetitiveness of repetitive negative thinking, intrusiveness of repetitive negative thinking, difficulties to disengage from repetitive negative thinking, unproductiveness of repetitive negative thinking and capturing mental capacity as well as higher levels of extreme focus on healthy eating.

The second objective of the present study was to identify the determinants of cognitive restraint among adults with varying dietary patterns. The results partially confirmed our hypotheses (H3 and H4) Regression analyses revealed that higher levels of the core characteristics of repetitive negative thinking (repetitiveness, intrusiveness, difficulties to disengage) predicted cognitive restraint in vegans. This means that perseverant thinking leads vegans to exacerbate their restricting food intake for purposes of weight control. It is worth adding that previous (experimental) study has shown that suppressing food-related thoughts can cause a subsequent increase in consumption relative to individuals not suppressing or thinking about food [52]. Participants showing high levels of dietary restraint in the suppression condition consumed significantly more chocolate than those showing high levels of dietary restraint in the expression or control condition. In addition, participants reporting frequent use of thought suppression reported greater chocolate cravings [52]. It can be presumed that vegans do not suppress their food-related thoughts and therefore they restrict the amount and quality of food consumed. The second explanation could be that enduring negative thoughts signal a cognitive conflict that leads to the disengagement of attention from negative thoughts via attentional control. From this perspective, repetitive negative thinking results from impaired cognitive conflict signaling and/or difficulties in enacting attentional control to divert attention away from one’s negative thoughts [53]. It is worth emphasizing that inducing repetitive negative thinking in non-clinical samples has been shown to negatively impact cognitive, behavioral, and interpersonal performance [54]. Our results demonstrate that repetitive negative thinking influences increased cognitive restraint in vegans. Positive feelings related to an extreme focus on healthy eating (orthorexia nervosa) were a significant predictor of cognitive restraint in vegetarians. Positive feelings related to orthorexia nervosa are characterized by: feeling in control, feeling a sense of satisfaction in eating healthily, and feeling great and peaceful after eating healthily [37]. There is increasing evidence that a daily diet rich in fruits and vegetables is associated with greater positive affect, positive mood, happiness, and life satisfaction [55]. Recent studies have suggested that high fruit and vegetable consumption may be a causal factor in promoting states of positive well-being and eudaemonic well-being as well [55,56]. Dietary cognitive restraint has been also shown to be positively associated with self-reported consumption of healthy foods [57]. Based on these results, it could be supposed that adhering to a vegetarian diet may be related with positive feelings and lead to conscious restriction of food intake in order to control body weight or to promote weight loss. Our results show that problems associated with healthy eating and feeling positively about healthy eating predicted cognitive restraint in individuals following an omnivorous diet. Problems associated with healthy eating are characterized by: often turning down social events that involve eating unhealthy food, following a diet with many rules, being distracted by thoughts of eating healthily, considering one’s own healthy eating as a source of stress in a relationship, having difficulty finding restaurants that serve healthy food and food restrictions [37]. We can suppose that in these individuals cognitive restraint can be offset by inappropriate eating patterns leading to increased orthorexia nervosa.

Some limitations of the present study should be noted. First, the cross-sectional nature of the data means no conclusion can be drawn regarding the causation of our findings. For investigating the causality, the future study should be focused on experimental and longitudinal studies. Second, there are unequal sample sizes across the plant-based diet groups. Greater numbers in each group (particularly in vegan group) would increase statistical power and the strength of conclusions. Third, body measurements are subjective. Although self-measurement values have been found to be valid and reliable when compared to direct measurements, discrepancies do still occur when those measurements are compared with objective measurement methods [58]. It is worth pointing out that previous results have shown that women and men have a tendency to overestimate their height and to underestimate their weight (the underestimation of weight is more pronounced in women [58]). Therefore, objectively measured body mass index should be considered to avoid inaccurate estimation of body weight and height. Finally, current sample data provide limited information on sociodemographic characteristics. More detailed information on sociodemographic characteristics of the samples (e.g., information on occupation, education, income, being pregnant) should be available for providing better knowledge of the sociodemographic profiles of self-reported vegetarians and vegans (nowadays there are few published studies on sociodemographic characteristics of those groups [59]).

## 5. Conclusions

Our results indicated that both vegetarian and vegan groups showed a significantly lower cognitive restraint, lower emotional eating, and lower uncontrolled eating than those who followed an omnivorous diet. Our results are compatible with findings demonstrating that individuals following a plant-based diet (vegetarians and vegans) show more cognitions, behaviours and feelings related to orthorexia nervosa than a group following an omnivorous diet.

This study was the first to explore predictors of conscious restriction of food intake in order to control body weight or to promote weight loss of individuals following different dietary patterns. Longitudinal examinations confirming our findings are needed. Nevertheless, knowledge of predictors of cognitive restraint (repetitive negative thinking and orthorexia nervosa) may serve as a psychological intervention goal or psychoeducation goal among individuals with varying dietary patterns. In addition, healthcare professionals should keep repetitive negative thinking and orthorexia nervosa status in mind when dealing with individuals adhering to a vegan and vegetarian dietary patterns. The development of interventions designed to reduce levels of negative thoughts among individuals with high levels of cognitive restraint could be helpful to end or struggle with perseverant thinking or cognitive distortions that can lead to disordered eating behaviours (e.g., orthorexia nervosa). The present study provides information about the determinants of the cognitive restraint by suggesting that this construct is linked with orthorexia nervosa among individuals with varying dietary patterns and with repetitive negative thinking among individuals following a vegan diet. Our findings concerning the predictors of cognitive restraint could lead to effective measures, e.g., for identifying at-risk individuals. Furthermore, our results could serve as a starting point for future studies.

There are a number of gaps in our knowledge around cognitive restraint. The existing research on cognitive restraint and varying dietary patterns (especially a vegetarian diet) is cross-sectional, so causality cannot be determined. Therefore, more research is needed in this area. Future research should track cognitive restraint and other disordered eating patterns among individuals varying dietary patterns longitudinally to determine cause and effect.

## Figures and Tables

**Table 1:** Cognitive restraint scores between vegetarians and omnivores: the inconsistent results.

Higher Cognitive Restraint Scores in Vegetarians	Higher Cognitive Restraint Scores in Non-Vegetarians	Not Difference in Cognitive Restraint between the Two Groups
Barr et al. [11] ** N vegetarians = 23 *N* non-vegetarians = 22 Method: The Three-Factor Eating Questionnaire	Brytek-Matera [12] * N vegetarians = 188 *N* non-vegetarians = 182 Method: The Three-Factor Eating Questionnaire (TFEQ-R18)	Barr and Broughton [13] * N current vegetarians = 90 *N* past vegetarians = 35 *N* non-vegetarians = 68 Method: Multiple-pass 24-h diet recall
Gilbody et al. [14] * N total = 131 young adults women Method: the Dutch Eating Behavior Questionnaire	Curtis and Comer [15] * N total = 90 female undergraduate students and community members Method: The Three-Factor Eating Questionnaire	Barthels et al. [16] * N vegetarians = 63 *N* vegans = 114 *N* non-vegetarians = 182 Method: the Restraint Eating Scale
Martins et al. [17] * N vegetarians = 70 *N* non-vegetarians = 105 *N* semi-vegetarians = 49 Method: The Three-Factor Eating Questionnaire	Janelle and Barr [18] * N vegetarians = 23 *N* non-vegetarians = 22 Method: The Three-Factor Eating Questionnaire	Brytek-Matera et al. [19] * N vegetarians = 39 *N* vegans = 40 *N* non-vegetarians = 174 Method: The Three-Factor Eating Questionnaire (TFEQ-R18)
McLean and Barr [20] * N total = 1350 female university students Method: The Three-Factor Eating Questionnaire		Fisak et al. [21] * N vegetarians = 52 *N* non-vegetarians = 204 Methods: the Dutch Eating Behavior Questionnaire and the Three-Factor Eating Questionnaire
Trautman et al. [22] * N total = 330 college students Method: the Dutch Eating Behavior Questionnaire		Forestell et al. [23] * N vegetarians = 55 *N* pesco-vegetarians = 28 *N* semi-vegetarians = 29 *N* flexitarians = 37 *N* non-vegetarians = 91 Method: The Three-Factor Eating Questionnaire
Worsley and Skrzypiec [24] * N total = 2000 senior secondary school students Method: the Food Frequency Questionnaire		

* Cross-sectional study; ** Longitudinal study.

**Table 2:** Characteristics of measurements used in the present study.

Questionnaire	Objective	Number of Items	Subscales	Internal Reliability: Cronbach’s α
The Polish version of the Three-Factor Eating Questionnaire [39]	Assessment of three different aspects of eating behaviours	18	1. Cognitive restraint-conscious restriction of food intake in order to control body weight or to promote weight loss. 2. Uncontrolled eating-tendency to eat more than usual due to a loss of control over intake accompanied by subjective feelings of hunger. 3. Emotional eating-inability to resist emotional cues, the tendency to eat in response to negative emotions.	Cognitive restraint: α = 0.78 Uncontrolled eating: α = 0.84 Emotional eating: α = 0.86
The Polish version of the Perseverative Thinking Questionnaire [40]	Assessment of repetitive negative thinking from a content-independent perspective	15	1. Core characteristics of repetitive negative thinking: (a) repetitiveness, (b) intrusiveness and (c) difficulties to disengagement. 2. Unproductiveness of repetitive negative thinking. 3. Capturing mental capacity.	Cronbach’s α = 0.64–0.92
The Polish version of the Eating Habits Questionnaire [19]	Assessment of cognitions behaviours and feelings related to an extreme focus on healthy eating, which has been called orthorexia nervosa	21	1. Knowledge of healthy eating. 2. Problems associated with healthy eating. 3. Feeling positively about healthy eating.	Knowledge of healthy eating: α = 0.81 Problems associated with healthy eating: α = 0.82 Feeling positively about healthy eating: α = 0.82

**Table 3:** Descriptive characteristics of the study population.

Variable	Vegan Diet *N* = 47	Vegetarian Diet *N* = 100	Omnivorous Diet *N* = 107
		Mean (SD)	
Age	30.61 (11.64)	28.39 (8.92)	28.72 (9.87)
Body mass index (kg/m^2^)	21.46 (4.10)	21.97 (2.88)	23.01 (5.35)
Following plant-based diet (in moths)	44.40 (62.30)	82.90 (102.36)	-
		*N* (%)	
Number of meals consumed per day			
1	0 (0.0)	0 (0)	2 (1.9)
2	2 (4.3)	4 (4)	5 (4.7)
3	16 (34.0)	20 (20)	27 (25.2)
4	19 (40.4)	38 (38)	49 (45.8)
5	9 (19.2)	30 (30)	20 (18.7)
More than 5	1 (2.1)	8 (8)	4 (3.7)
Daily water intake			
Less than 1 L	2 (4.2)	2 (2)	18 (16.8)
From 1 L to 1,4 L	7 (14.9)	22 (22)	40 (37.4)
1,5 L	7 (14.9)	29 (29)	18 (16.8)
From 1,6 L to 1,9 L	4 (8.5)	3 (3)	3 (2.8)
2 L	15 (31.9)	25 (25)	21 (19.6)
From 2,1 L to 2,4 L	2 (4.3)	3 (3)	0 (0)
2,5 L	1 (2.1)	9 (9)	3 (2.8)
3 L	6 (12.8)	4 (4)	3 (2.8)
3,5 L	0 (0)	1 (1)	1 (0.9)
4 L	3 (6.4)	2 (2)	0 (0.0)
Dietary supplement consumption			
Yes	34 (72.3)	59 (59)	25 (23.4)
No	13 (27.7)	41 (41)	82 (76.6)
Type of supplement consumed (multiple choice question)			
Vitamin D	21 (44.7)	32 (32)	12 (11.2)
Vitamin B12	29 (61.7)	34 (34)	4 (3.7)
Vitamin C	1 (2.1)	10 (10)	2 (1.9)
Magnesium	4 (8.5)	13 (13)	8 (7.5)
Moderate-intensity physical activity			
Never	0 (0)	0 (0)	9 (8.4)
One per week	5 (10.6)	6 (6)	42 (39.2)
Twice per week	0 (0)	13 (13)	0 (0)
3 per week	7 (14.9)	25 (25)	23 (21.5)
5 per week	10 (21.3)	18 (18)	14 (13.1)
Every day	25 (53.2)	38 (38)	19 (17.8)
Vigorous-intensity Physical Activity			
Never	16 (34.04)	44 (44)	51 (47.7)
One per week	15 (31.91)	30 (30)	37 (34.6)
Twice per week	0 (0)	0 (0)	0 (0)
3 per week	10 (21.30)	20 (20)	14 (13.1)
5 per week	4 (8.51)	6 (6)	4 (3.7)
Every day	2 (4.30)	0 (0)	1 (0.9)
Main reason why participants decided to follow a plant-based diet			Not applicable
Animal welfare	25 (53.20)	60 (60)	
Health	10 (21.30)	10 (10)	
Ethics	4 (8.51)	9 (9)	
Care for the natural environment	2 (4.25)	9 (9)	
Religion	1 (2.12)	0 (0)	
Economic considerations	1 (2.12)	0 (0)	
Weight loss	2 (4.25)	0 (0)	
Other reason	2 (4.25)	12 (12)	
Main reason why participants are following a plant-based diet			Not applicable
Animal welfare	29 (61.6)	53 (53)	
Health	6 (12.8)	13 (13)	
Ethics	6 (12.8)	17 (17)	
Care for the natural environment	3 (6.4)	7 (7)	
Religion	1 (2.1)	0 (0)	
Other reason	2 (4.3)	10 (10)	

**Table 4:** The mean (M) (and standard deviation; SD) eating behaviours, orthorexia nervosa and repetitive negative thinking across the dietary patterns.

Variable	Vegan Diet (*N* = 47)		Vegetarian Diet (*N* = 100)		Omnivorous Diet (*N* = 107)		Vegan vs. Vegetarian Diet	Vegan vs. Omnivorous Diet	Vegetarian vs. Omnivorous Diet	Vegan vs. Vegetarian vs. Omnivorous Diet
	M (SD)	95% CI [LL, UL]	M (SD)	95% CI [LL, UL]	M (SD)	95% CI [LL; UL]	*p*-Value	*p*-Value	*p*-Value	*p*-Value
Cognitive restraint	5.70 (3.54)	0.51, 4.66	6.81 (3.58)	0.35, 6.09	8.64 (3.86)	0.37, 7.90	0.275	**0.000**	**0.001**	**0.000**
Uncontrolled eating	5.48 (5.10)	0.74, 3.99	7.34 (5.22)	0.52, 6.30	12.39 (7.08)	0.68, 11.03	0.275	**0.000**	**0.001**	**0.000**
Emotional eating	2.38 (2.82)	0.41, 1.55	2.85 (2.70)	0.27, 2.31	4.71 (3.17)	0.30, 4.11	1.00	**0.000**	**0.001**	**0.000**
Core characteristics of repetitive negative thinking	22.57 (8.57)	20.05, 25.09	23.85 (8.79)	22.10, 25.59	23.25 (9.09)	0.87, 21.50	1.00	1.00	1.00	0.709
Unproductiveness of repetitive negative thinking	6.57 (2.77)	0.40, 5.75	6.73 (2.69)	0.26, 6.19	7.16 (2.80)	0.27, 6.63	1.00	1.00	1.00	0.362
Capturing mental capacity	6.40 (3.31)	0.48, 5.43	6.76 (2.80)	0.28, 6.20	6.85 (3.10)	0.30, 6.26	1.00	0.659	0.763	0.688
Knowledge of healthy eating	14.38 (3.28)	0.47, 13.41	12.48 (2.63)	0.26, 11.95	10.13 (3.23)	0.31, 9.51	**0.001**	**0.000**	**0.000**	**0.000**
Problems associated with healthy eating	22.87 (4.76)	0.69, 21.47	20.24 (4.03)	0.40, 19.43	17.30 (5.49)	0.53, 16.25	**0.007**	**0.000**	**0.000**	**0.000**
Feeling positively about healthy eating	11.40 (2.80)	0.40, 10.58	10.81 (2.65)	0.26, 10.28	9.66 (2.75)	0.26, 9.13	0.656	**0.001**	**0.008**	**0.000**

M: mean; SD: standard deviation; 95% CI [LL, UL]: 95% confidence interval [LL: lower limit of a confidence interval, UL: upper limit of a confidence interval]. Post hoc tests with Bonferroni correction were used. Significant *p*-values are shown in boldface type (*p* < 0.05).

**Table 5:** Relationships among variables across the dietary patterns.

Variable	Vegan Diet (*N* = 47)		Vegetarian Diet (*N* = 100)		Omnivorous Diet (*N* = 107)	
	Cognitive Restraint					
	*r*	*p*-Value	*r*	*p*-Value	*r*	*p*-Value
Core characteristics of repetitive negative thinking	**0.325**	0.026	−0.62	0.537	**0.241**	0.013
Unproductiveness of repetitive negative thinking	0.102	0.497	0.003	0.976	**0.192**	0.048
Capturing mental capacity	0.036	0.808	−0.037	0.716	**0.214**	0.027
Knowledge of healthy eating	0.051	0.733	**0.288**	0.004	**0.461**	0.000
Problems associated with healthy eating	**0.336**	0.021	**0.273**	0.006	**0.505**	0.000
Feeling positively about healthy eating	0.266	0.70	**0.393**	0.000	**0.507**	0.000

Significant values of the correlation coefficients (*r*) are shown in boldface type (*p* < 0.05).

**Table 6:** Determinants of cognitive restraint in adults with varying dietary patterns.

**Cognitive Restraint among Vegans**	***ß***	***t***	***p*-Value**	***R*^2^**	**Δ*R*^2^**
Core characteristics of repetitive negative thinking	**1.004**	3.763	0.001	0.364	0.269
Unproductiveness of repetitive negative thinking	−0.460	−1.575	0.123		
Capturing mental capacity	−0.492	−1.981	0.055		
Knowledge of healthy eating	−0.051	−0.359	0.721		
Problems associated with healthy eating	0.111	0.718	0.477		
Feeling positively about healthy eating	0.254	1.148	0.146		
**Cognitive restraint among vegetarians**	***ß***	***t***	***p*-Value**	***R*^2^**	**Δ*R*^2^**
Core characteristics of repetitive negative thinking	−0.114	−0.723	0.471	0.191	0.139
Unproductiveness of repetitive negative thinking	0.145	0.997	0.331		
Capturing mental capacity	−0.030	−0.185	0.854		
Knowledge of healthy eating	0.115	1.052	0.296		
Problems associated with healthy eating	0.100	0.907	0.367		
Feeling positively about healthy eating	**0.310**	2.874	0.005		
**Cognitive restraint among non-vegetarians**	***ß***	***t***	***p*-Value**	***R*^2^**	**Δ*R*^2^**
Core characteristics of repetitive negative thinking	0.068	0.395	0.694	0.364	0.325
Unproductiveness of repetitive negative thinking	0.005	0.035	0.972		
Capturing mental capacity	0.084	0.541	0.590		
Knowledge of healthy eating	0.182	1.510	0.134		
Problems associated with healthy eating	**0.241**	2.294	0.024		
Feeling positively about healthy eating	**0.243**	2.142	0.035		

Significant *β*’s are shown in boldface type (*p* < 0.05). Participants following a vegan diet: *F* change (6,40) = 3.818, *p* = 0.004; participants following a vegetarian diet: *F* change (6,93) = 3.657, *p* = 0.003; participants following an omnivorous diet: *F* change (6,100) = 9.519, *p* = 0.000. *ΔR*^2^: the change in *R*^2^.
